# Lipid metabolism in B cell biology

**DOI:** 10.1002/1878-0261.13560

**Published:** 2023-12-11

**Authors:** Rens Peeters, Julia Jellusova

**Affiliations:** ^1^ School of Medicine and Health, Institute of Clinical Chemistry and Pathobiochemistry Technical University of Munich Germany; ^2^ TranslaTUM, Center for Translational Cancer Research Technical University of Munich Germany

**Keywords:** B cell development, B cell malignancies, B cells, immunometabolism, lipid metabolism, lipid signalling

## Abstract

In recent years, the field of immunometabolism has solidified its position as a prominent area of investigation within the realm of immunological research. An expanding body of scientific literature has unveiled the intricate interplay between energy homeostasis, signalling molecules, and metabolites in relation to fundamental aspects of our immune cells. It is now widely accepted that disruptions in metabolic equilibrium can give rise to a myriad of pathological conditions, ranging from autoimmune disorders to cancer. Emerging evidence, although sometimes fragmented and anecdotal, has highlighted the indispensable role of lipids in modulating the behaviour of immune cells, including B cells. In light of these findings, this review aims to provide a comprehensive overview of the current state of knowledge regarding lipid metabolism in the context of B cell biology.

## Graphical abstract

Lipids play a diverse but important role in B cell biology. They serve as fuel for energy production, affect transcription factors and epi‐genetic modifications, and are important for signalling and in post‐translational protein modifications. In this review, we summarize the current knowledge on lipid metabolism in B cells throughout development and during malignant transformation.

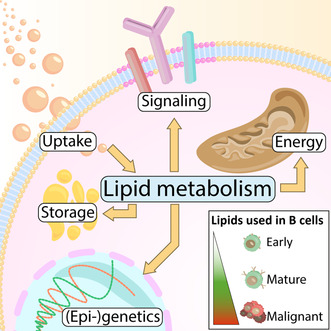

**Abbreviations**
AA: arachidonic acid
ACC: acetyl‐CoA carboxylase
ACLY: ATP citrate lyase
ACSL1: acetyl‐CoA synthetase long chain family member 1
ADP: adenosine diphosphate
ATP: adenosine triphosphate
BCR: B cell receptor
BL: Burkitt lymphoma
CiC: citrate carrier
CLL: chronic lymphocytic leukaemia
CoA: coenzyme A
CPT1/2: carnitine palmitoyl transferase 1/2
DAG: diacylglycerol
DHA: docosahexaenoic acid
DLBCL: diffuse‐large B cell lymphoma
DZ: dark zone
EBV: Epstein–Barr virus
EPA: eicosapentaenoic acid
ER: endoplasmatic reticulum
ETC: electron transport chain
FA: fatty acid
FABP: fatty acid binding protein
FADH2: flavine‐adenine‐dinucleotide
FAO: fatty acid oxidation
FASN: fatty acid synthase
FATP: fatty acid transporter protein
FPP: farnesyl pyrophosphate
G3P: glyceraldehyde 3‐phosphate
GC: germinal centre
GGPP: geranylgeranyl diphosphate
GLUT1: glucose transporter 1
GSK3: glycogen synthase kinase 3
HDAC: histone deacetylase
HGAL: human germinal centre‐associated lymphoma
HIF1a: hypoxia‐inducible factor 1a
HMG‐CoA: hydroxy‐3‐methylglutaryl‐CoA
IP3: inositol triphosphate
LCFA: long‐chain fatty acid
LPL: lipoprotein lipase
LXR: liver X receptor
LZ: light zone
MAG: monoacylglycerol
MCFA: medium‐chain fatty acid
mTOR: mammalian target of rapamycin
MUFA: monounsaturated fatty acid
MZ: marginal zone
NADH: nicotinamide adenine dinucleotide
NADPH: nicotinamide adenine dinucleotide phosphate
OA: oleic acid
OxPhos: oxidative phosphorylation
PA: palmitic acid
PC: plasma cell
PI3K: phosphoinositide 3‐kinase
PIP2: phosphatidylinositol 4,5‐bisphosphate
PIP3: phosphatidylinositol (3,4,5)‐trisphosphate
PKC: protein kinase C
PLC: phospholipase c
PO: palmitoleic acid
PS: phosphatidylserine
PUFA: polyunsaturated fatty acid
ROS: reactive oxygen species
SCD: stearoyl‐CoA desaturase
SCFA: short‐chain fatty acid
SFA: saturated fatty acid
SHM: somatic hypermutations
TAG: triacylglycerol
TCA: tricarboxylic acid
VLCFA: very‐long‐chain fatty acid

## 1 Introduction

Recent years have brought a growing appreciation of how metabolism shapes immune cell activity. Considering the remarkable mobility and functional heterogeneity of immune cells, it is perhaps not surprising that these cells require a high level of metabolic flexibility unmatched by other, less dynamic types of cells. Within seconds, an immune cell has to be able to switch from relative quiescence to a highly activated state. The metabolic program is rapidly altered to support the cells' new function. Initial metabolic adaptations can occur within minutes of stimulation [1] and dynamically support different phases of activation, cell cycle progression and differentiation [2]. Moreover, immune cells inhabit a variety of niches which can profoundly differ in their metabolic composition, thereby shaping cellular metabolic programs.

B cells, together with T cells provide the immune system with specific recognition and a tailor‐made response to virtually every pathogen [3]. To accomplish this task, B cells go through several phases of gene rearrangements, mutations [4], selection [5, 6], proliferative expansion [7] and relative quiescence [8] during their life span. B cells are characterized by a semi‐nomadic life and can be found in metabolically heterogeneous environments such as the spleen, bone marrow, gut, peritoneal cavity and blood. For these reasons, metabolic demands of various B cell subsets differ substantially [9]. Aberrant control of metabolic fate has been associated with a plethora of B cell‐derived pathologies ranging from autoimmunity [10, 11] to B cell malignancies [12, 13, 14]. Other great reviews [9, 15, 16] have covered our general knowledge on the metabolic regulation in normal and aberrant B cells and highlighted not only the importance of metabolic reprogramming to meet energetic and biosynthetic needs of different B cell subsets, but have also underscored the diverse spectrum of roles metabolites can play outside of bioenergetics. However, the major focus in the field of B cell metabolism to date has been on glucose metabolism, with less attention being paid to other metabolites. Lipids not only play an important role in bioenergetics but can also shape cell signalling. Here, we summarize what is currently known about the role of lipids in different B cell subsets and discuss outstanding challenges and possible future directions in the field.

## 2 Lipid metabolism

**Fig. 1:**
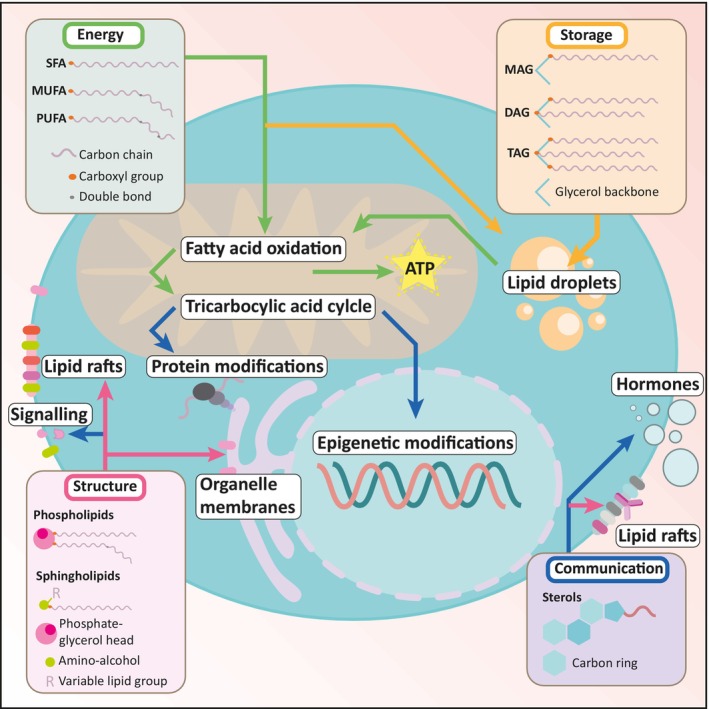
Overview of the different lipid classes and their roles in B cells. SFA, MUFA, and PUFA are used in energy (ATP) generation via FAO in mitochondria. Breakdown products of FAO fuel the TCA cycle. TCA intermediates can be used for protein modifications and epigenetic modifications. The FAs can also be processed for storage in lipid droplets. This happens by linking the FAs to a glycerol backbone, creating monoacylglycerol (MAG), diacylglycerol (DAG) and TAG. Reverse reactions can also free up fatty acids from TAG to fuel mitochondrial FAO again. Phospholipids, sphingolipids and sterols such as cholesterol are lipids with more complex head‐groups and are important membrane integrity and fluidity. They can form lipid rafts or can be modified, thereby aiding communication by facilitating signalling domains or via second messengers. Green arrows: Lipids involved in energy homeostasis. Orange arrows: Lipids involved in storage. Magenta arrows: Lipids involved in structure. Blue arrows: Lipids involved in communication.

Lipid metabolism refers to the biochemical processes involved in the synthesis, breakdown, and utilization of lipids (fats) within an organism or within a cell. Lipids form the structural components of cell membranes, can function as signalling molecules, have a role in redox balance and are the main contributors to long‐term energy storage (Fig. 1). Lipid metabolism is crucial for both B and T cells in adaptive immunity [17]. B cells rely on lipids for membrane synthesis, especially phospholipids, and lipid rafts play a role in signalling and antibody production during activation. T cells, undergoing metabolic reprogramming upon activation, prioritize glycolysis over oxidative phosphorylation (OxPhos) [18]. While both cell types utilize lipids for membrane structure and signalling, their specific metabolic profiles differ, reflecting their distinct roles in humoral and cell‐mediated immunity, respectively [19]. In the next section, we outline these different lipid species and what role they play in B cell biology.

### 2.1 Lipid classes

The term lipid refers to a diverse group of organic molecules which are loosely characterized by their insolubility in water. Some of the main classes of lipids relevant to B cell biology are:

1. Fatty acids: Fatty acids (FAs) consist of varying lengths of hydrocarbons with a carboxyl group at one end (Fig. 1). According to their length, FAs can be classified into short‐chain fatty acids (SCFA), medium‐chain fatty acids (MCFA), long‐chain fatty acids (LCFA), and very long chain fatty acids (VLCFA).
  Fatty acids can be saturated, meaning there are no double bonds within the carbohydrate chain, or unsaturated, which harbour one or more double bonds within the carbohydrate chain. Polyunsaturated fatty acids (PUFAs) with two or more double bonds such as arachidonic acid, eicosapentaenoic acid (EPA), docosahexaenoic acid (DHA) have two main roles: as precursors of signalling molecules and as building blocks of membrane lipids. PUFAs can also give rise to lipid mediators such as lipoxins, resolvins, protectins, and maresins which play a role in the resolution phase of inflammation [20] and have been reported to enhance B cell differentiation to plasma cells (PCs) [21]. Monounsaturated fatty acids (MUFAs) such as oleic acid (OA) and palmitoleic acid (PO) have been reported to support mitochondrial metabolism and mammalian Target of Rapamycin C1 (mTORC1) activity in B cells [22]. In general, FAs serve in energy production or as precursors for more complex lipids but can also affect cell signalling via post‐translational protein modifications or by altering enzyme activity.
2. Phospholipids: Phospholipids are a major component of cell membranes (Fig. 1). They consist of a glycerol molecule bonded to two FAs and a phosphate group. The amphipathic nature of phospholipids allows them to form a lipid bilayer, serving as the structural foundation of cell membranes. It is important to note that the exact composition of phospholipids can alter physical properties of plasma membranes. PUFAs in glycerophospholipids display a higher structural plasticity and increase membrane fluidity in comparison to saturated fatty acids (SFAs) or MUFAs [23]. Additionally, phospholipids are essential for the formation of lipid rafts, specialized membrane domains crucial for B cell signalling. Moreover, phospholipid headgroups modulate downstream pathways, affecting calcium signalling and protein kinase C (PKC) activation [24]. As B cells undergo development, activation, and antibody production, the synthesis and regulation of phospholipids play a central role in supporting these dynamic processes and ensuring the overall functionality of B cells in the immune response.
3. Sphingolipids: Sphingolipids are another component of cell membranes (Fig. 1). They are characterized and named after their sphingoid base backbone; an amino alcohol. They can be involved in many cellular functions such as signalling, adhesion, apoptosis, survival and inflammation [25]. Sphingolipids play a crucial role in various aspects of the immune system, including in B cell biology [26]. They contribute to the structure and fluidity of B cell membranes, play a key role in the formation of lipid rafts, and are integral to B cell receptor (BCR) signalling pathways [27]. Sphingolipids influence cell survival, apoptosis, and immunological synapse formation during interactions with other immune cells. Additionally, they participate in antigen presentation, impacting the efficiency of B cell responses [28]. In germinal centres (GCs), sphingolipids likely regulate signalling cascades affecting B cell selection and differentiation. Sphingosine‐1‐phosphate (S1P), a sphingolipid, guides B cell migration within lymphoid organs [29].
4. Glycolipids: Glycolipids are lipids that have a carbohydrate group attached to them. They are present on the outer surface of cell membranes and are involved in cell recognition and cell signalling. Glycolipids play important roles in processes like cell adhesion, immune response, and cell–cell communication [30]. The presence and regulation of glycolipids are integral to the dynamic processes of B cell activation, differentiation, and their participation in immune functions.
5. Steroids: Steroids are lipids characterized by a structure of four fused carbon rings (Fig. 1). Cholesterol is a well‐known steroid that plays a crucial role in cell membranes by restricting their fluidity and stability. Steroids are also precursors for many different signalling molecules [31]. Glucocorticoid steroids, such as cortisol, can have immunosuppressive effects on B cells. They inhibit the proliferation of B cells and suppress the production of antibodies [32, 33]. This immunosuppressive action is often utilized in therapeutic settings to dampen immune responses, as seen in the use of corticosteroids to treat autoimmune conditions or to manage inflammatory responses [34]. While steroids can impact B cell function, their effects are broad, affecting various aspects of the immune system [35].
6. Triacylglycerols: Triacylglycerols (TAG), also known as triglycerides, are a primary form of energy storage in cells (Fig. 1). They consist of glycerol bonded to three FAs. TAGs are stored in specialized organelles called lipid droplets and can be broken down when energy is needed, providing a concentrated and flexible source of fuel for cellular processes. This dynamic regulation of TAG metabolism provides B cells with the necessary energy substrates for their metabolic needs during immune responses, contributing to the overall functionality of B cells [36, 37].
7. Immunoxysterols: Immunoxysterols are a class of molecules derived from cholesterol metabolism that play a role in immune regulation. The term specifically refers to oxidized derivatives of cholesterol, known as oxysterols. Oxysterols are formed through enzymatic or non‐enzymatic oxidation of cholesterol, and they act as signalling molecules that influence various aspects of the immune system. Research suggests that oxysterols may influence B cell functions such as migration, proliferation, differentiation into antibody‐secreting cells, and antibody class switching [38, 39]. Oxysterols can act as ligands for certain nuclear receptors, including the liver X receptors (LXRs), which are involved in the regulation of cholesterol metabolism and immune responses [40].

### 2.2 Fatty acid uptake and metabolism

Most lipids can be synthesized directly (TAGs, phospholipids) or indirectly (sterols, via acetyl‐CoA) from FAs. As such, FAs play a crucial role in adaptive immunity by serving as essential components for various cellular processes in immune cells. These lipids are fundamental for energy production, membrane structure, and signalling pathways during the activation and function of immune cells, particularly B and T lymphocytes. The dynamic regulation of FA metabolism is vital for supporting the increased energy demands and biosynthetic requirements associated with the rapid proliferation, differentiation, and effector functions of immune cells during adaptive immune responses. Another recent review has expertly highlighted the similarities and differences between B and T in FA utilization [17]. In this section, we summarize our current knowledge on FA utilization in B cells.

**Fig. 2:**
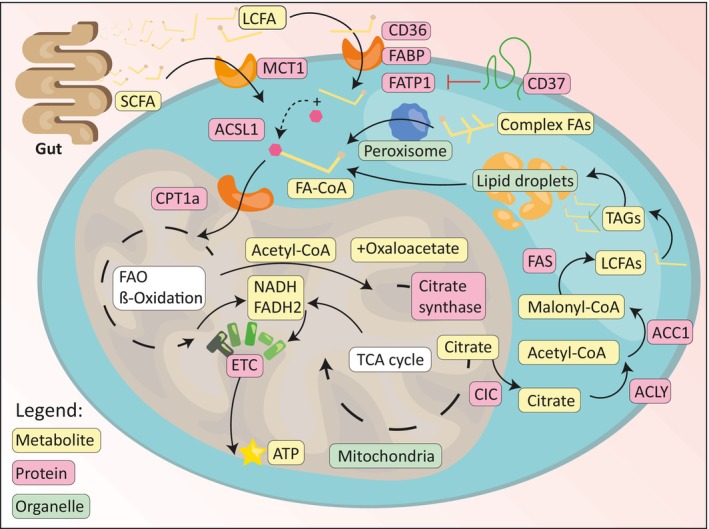
Overview of metabolic pathways involved in B cell lipid and energy homeostasis. SCFA and LCFA are taken up by B cells via specific transporters. SCFAs are taken up via MCT1. LCFAs are taken up in a variety of ways, by scavenger receptor CD36, FABPs and FATPs. FATP1 is inhibited by tetraspanin CD37, whereas mechanisms of inhibition of the other transporters remain elusive. Once inside, the fatty acids are activated by means of coenzyme A (CoA) tethering, executed by acetyl‐CoA synthase 1 (ACSL1). Activated fatty acids (FA‐CoA) can be transported into the mitochondria via carnitine palmitoyl‐transferase 1a (CPT1a). Here, the FAs are broken down in an enzymatic cyclical cascade called FAO or ß‐oxidation. Resulting metabolites nicotinamide‐adenine‐dinucleotide (NADH) and flavin adenine dinucleotide (FADH_2_) can be oxidized by the ETC in order to synthesize ATP. Moreover, two‐carbon acetyl‐CoA is used in the TCA cycle to generate intermediates, and more NADH and FADH2. Alternatively, citrate can be formed by combining acetyl‐CoA and oxaloacetate by citrate synthase. The resulting citrate can be carried into the cytosol by CIC. Here ACLY can free up acetyl‐CoA again from the metabolite. ACC1 can subsequently activate this into malonyl‐CoA, the precursor for fatty acid synthesis by FAS. Three newly produced LCFAs can be conjugated to the carbohydrate G3P in order to form TAGs. These can then be stored in specialized organelles called lipid droplets. More complex branched‐chain fatty acids are first simplified in peroxisomes, releasing acyl‐CoAs for further use. Whenever needed, acyl‐CoAs can be freed‐up from lipid droplets.

Short‐chain fatty acids such as acetate, propionate and butyrate are primarily produced by commensal microbiota [41]. They can be taken up by specialized SCFA transporters on B cells such as monocarboxylate transporter 1 (MCT1) [13, 42] and enter the cytosol (Fig. 2). Similarly, LCFAs are obtained from dietary sources, but some can be synthesized de novo [43]. Cellular uptake is facilitated by the scavenger receptor CD36 aided by small intracellular lipid chaperones called fatty acid‐binding proteins (FABPs) [44]. Fatty acids can also be taken up via other transporters such as fatty acid transporter proteins (FATPs) (Fig. 2) [45]; however, their expression pattern and exact role in B cells are currently poorly understood. Once inside the cytosol, SCFAs and LCFAs become activated by means of co‐enzyme A (CoA) addition (Fig. 2) [46]. This step is required for further processing of the FAs. Consensus is lacking on whether this activation step is an intrinsic function of some of the transporter proteins or whether specialized acyl‐CoA synthases such as ACSL1 must always be present (Fig. 2). Overexpression of ACSL1 has been shown to drive increased fatty acid oxidation (FAO), thus supporting the latter scenario [47]. Moreover, inhibition of ACSL1 in macrophages results in a marked reduction of CD36 expression, reinforcing the notion that the activation and uptake are separate processes, yet intricately related to each other [47, 48].

To overcome the LCFA‐impermeable mitochondrial membrane, transport of activated LCFAs from the cytosol to the mitochondria is dependent on the carnitine shuttle system composed of carnitine‐palmitoyl transferase 1 and 2 (CPT1 and CPT2) and carnitine‐acylcarnitine translocase. SCFAs can enter the mitochondria independently of the carnitine system [49]. During FAO high‐energy molecules such as NADH and FADH_2_ are produced via a cyclic series of enzymatic reactions known as FAO or ß‐oxidation, with the ‘ß’ referring to the second‐to‐last carbon atom of the acyl‐chain which is cleaved off in each cyclic step. The resulting two‐carbon molecule, mitochondrial acetyl‐CoA, can subsequently enter the citric acid cycle (also known as the Krebs cycle or tricarboxylic acid, TCA cycle) to generate TCA intermediates and energy in the form of adenosine triphosphate (ATP) [50].

B cells can also synthesize lipids *de novo* in a process known as lipogenesis. FA synthesis starts within the mitochondria by Citrate Synthases combining acetyl‐CoA moieties and oxaloacetate into citrate (Fig. 2). Mitochondrial citrate carriers (CICs) can transport the citrate from the mitochondrial matrix into the cytosol. Here, ATP citrate lyase (ACLY) splits it back into oxaloacetate and acetyl‐CoA [51]. It might seem odd that acetyl‐CoA is not transported directly, but the non‐depolarized mitochondrial membrane is impermeable to acetyl‐moieties, although non‐specific release of acetyl‐CoA via “permeability transition pore complexes” has been observed before and warrants further investigation [52]. Mitochondrial membrane impermeability for acetyl‐CoA allows for spatiotemporal separation of inherently different processes using the same metabolite. The cytosolic acetyl‐CoA is a two‐carbon moiety that has to be activated in order to be elongated into a LCFA. This irreversible activation step is carried out by an enzyme called acetyl‐CoA carboxylase (ACC1), which combines bicarbonate with acetyl‐CoA at the cost of one ATP into malonyl‐CoA (Fig. 2) [53]. A dimerized multi‐unit protein complex containing seven catalytic domains, collectively known as the fatty acid synthase (FAS), uses malonyl‐CoA to elongate acetyl‐CoA in condensation steps of two carbons. This cyclical elongation continues at the cost of NADPH until there is a 16‐carbon acyl‐chain known as palmitic acid (PA). Acylation of glycerol‐3‐phosphate (G3P, generated via glycolysis) with three individual chains of PA results in TAGs, the main lipid species used for storage in lipid droplets (Fig. 2) [54]. When energy demands increase, stored triglycerides are hydrolysed, releasing FAs to meet the energy requirements of the cell, thereby providing great metabolic flexibility to the B cell [55]. Intermediates of the TAG synthesis or breakdown cascades may serve as activators or inhibitors of signalling pathways controlled by peroxisome proliferator‐activated receptor‐γ (PPARγ), the mTOR, or PKC isoforms, all important players in B cell biology [56, 57, 58]. Alternative to mitochondrial processing, FAs can also be oxidized in peroxisomes to generate acyl‐CoA or acetyl‐CoA without ATP generation. Peroxisomes primarily process more complex FAs such as VLCFAs (22 carbons or longer) or mono/poly‐branched FAs [59]. This process is believed to free up relatively simple acyl‐CoAs for the mitochondria, which can in turn use these to generate TCA intermediates or energy [60].

## 3 Lipids in B cell signalling

Lipids profoundly impact B cell signalling through direct roles as signalling molecules and indirect modulation of cellular processes. As signalling molecules, lipids can directly engage receptors and activate intracellular pathways. Indirectly, they influence membrane fluidity and lipid rafts, crucial for BCR signalling, and affect transcription by participating in intricate regulatory networks.

### 3.1 Lipids as signalling molecules

**Fig. 3:**
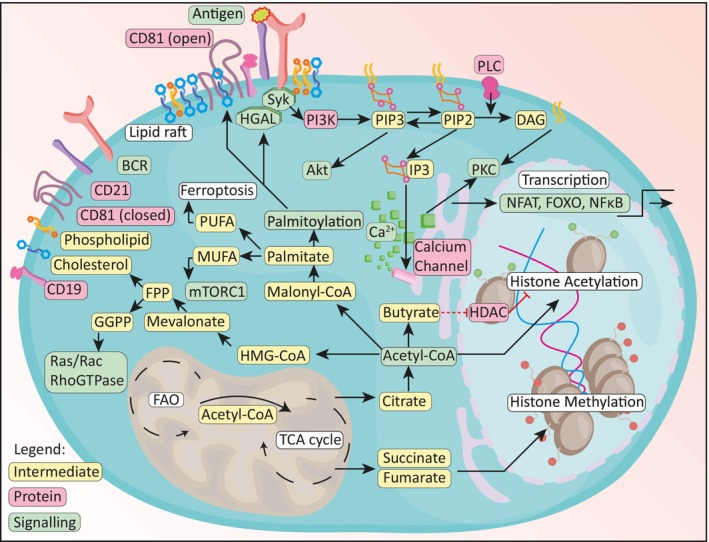
Overview of lipids involved in B cell signalling pathways. Membrane proteins CD19 and CD21 are brought in proximity to the BCR by tetraspanin CD81 via lipid rafts containing cholesterol and phospholipids upon antigen recognition. This allows tyrosine‐protein kinase Syk to activate PI3K, which phosphorylates PIP2 into PIP3. PIP3 recruits and activates protein kinase B (Akt). PIP2 can be cleaved by phospholipase C (PLC) into diacylglycerol (DAG) and inositol 1,4,5‐triphosphate (IP3) [61, 62]. IP3 binds to calcium channels on the ER to release calcium (Ca^2+^). Both the freed DAG and the released calcium activate PKC [61, 62]. Ultimately, activation of these signalling pathways leads to translocation of transcription factors such as NFAT, FOXO and NF‐κB. Moreover, PKC signalling induces mitochondrial remodelling and prepares the B cells for PC differentiation [63]. In the mitochondria, FAO provides acetyl‐CoA for the TCA cycle. TCA intermediates succinate and fumarate inhibit histone demethylation [69]. TCA intermediate citrate is used to generate cytosolic acetyl‐CoA, which can be used to synthesize butyrate, which potentially inhibits histone deacetylation (HDAC), or is directly used for histone acetylation. Alternatively, cytosolic acetyl‐CoA is used to synthesize either 3‐hydroxy‐3‐methylglutaryl‐CoA (HMG‐CoA) or malonyl‐CoA. The former is an intermediate in the mevalonate pathway, which produces FPP as precursor for either cholesterol or GGPP, which in turn modulates activity of Ras, Rac and RhoGTPases. Malonyl‐CoA is a precursor for fatty acid palmitate synthesis, which in turn can be desaturated into MUFAs or PUFAs which affect mTORC1 signalling or ferroptosis respectively. Alternatively, palmitate is used for palmitoylation of proteins CD81 or human germinal centre‐associated lymphoma (HGAL), thereby increasing their activity.

Specific lipids such as phosphatidylinositol phosphates recruit signalling proteins with lipid‐binding domains to the plasma membrane. For example, the kinase Akt harbours a pleckstrin homology domain which binds to phosphatidylinositol 3,4,5‐triphosphate (PIP3) in order to induce downstream signalling (Fig. 3). Lipids are also incorporated into signalling pathways and act as second messengers. For instance, the hydrolysis of PIP2 into IP3 and DAG is a central event of BCR induced signalling and necessary to promote calcium release from the endoplasmic reticulum (ER; Fig. 3). The resulting calcium release allows PKC to bind to, and be activated by DAG [61, 62], which in turn has been shown to dictate B cell fate via metabolic reprogramming [63]. Furthermore, sphingolipids such as sphingosine‐1‐phosphate (S1P) can directly activate specific G protein coupled receptors, whose downstream signalling in turn affect lymphocyte migration, inflammation and survival [64]. Moreover, eicosanoids like prostaglandins, leukotrienes and thromboxanes have been reported to directly affect the inflammatory status of lymphocytes [65].

### 3.2 Lipids as membrane modulators

Lipids play an important role not only in cellular bioenergetics, but can also shape cell fate decisions by modulating cell signalling by altering membrane composition (Fig. 3). Upon activation, B cells profoundly alter the composition, distribution and dynamics of membrane lipids [27, 66]. These changes determine how signals from cell surface receptors are transmitted. The plasma membrane contains various lipid molecules, which form dynamic microdomains and act as signalling platforms. The so‐called lipid rafts, rich in cholesterol and glycosphingolipids, play an important role in BCR activation and its interaction with co‐receptors [27, 28, 66]. Lipid rafts can facilitate proximation of signalling molecules in so‐called nanoclusters, thereby enhancing their downstream signal transduction [67]. Interestingly, cholesterol content of the plasma membrane changes during B cell maturation. Unlike mature B cells, transitional immature B cells display relatively low levels of cholesterol resulting in impaired compartmentalization of the BCR into cholesterol‐enriched domains upon BCR engagement. This reduces their ability to sustain certain aspects of BCR signalling [68].

### 3.3 Lipids in (epi‐)genetic regulation

Downstream of the lipid signalling pathways via PIP3 and Ca^2+^, transcription factors like Nuclear Factor of Activated T cells (NFAT), Forkhead Box O (FOXO) and Nuclear Factor‐kappa B (NF‐κB) are primed to translocate to (NFAT and NF‐κB) or away from the nucleus (FOXO). These transcription factors regulate the transcription of genes affecting B cell activation, response to antigen, proliferation, cell cycle arrest, class switch recombination, somatic hypermutations (SHMs) and affinity maturation (Fig. 3) [70, 71, 72]. Lipids can also affect cell fate on the level of epigenetic modulation. FAO‐derived acetyl‐CoA can be used as a substrate for histone acetyltransferases to promote histone acetylation [73]. Histone acetylation is a common epigenetic modification, that alters chromatin architecture and thereby gene expression. In addition to shaping substrate availability for epigenetic modifications, FAs can also regulate the activity of enzymes involved in epigenetic reprograming. SCFAs such as butyrate have been shown to inhibit the activity of histone deacetylases (HDACs) (Fig. 3) [74]. Two partially conflicting studies report on the effect of SCFAs in B cell chromatin remodelling and function. One study suggests that SCFAs boost B cell metabolism but also suppress HDAC activity and induce expression of genes necessary for PC differentiation [75]. The second study proposes the opposite‐ a reduction of PC differentiation upon SCFAs feeding [76]. The authors of the latter study argue that the SCFA concentration used in experiments strongly impacts functional outcome and low vs high concentrations of SCFAs can result in opposing phenotypes.

### 3.4 Lipids in post‐translational protein modification

Lipids can also affect cell signalling via post‐translational protein modification. Certain lipid metabolites can be post‐translationally attached to proteins to alter protein stability, or localization to specific cellular compartments or membrane subdomains. Post‐translational modifications which include lipids are for example protein prenylation, palmitoylation and myristoylation [77]. Protein S Acylation (or S‐palmitoylation, in this text referred to as palmitoylation) is a common and reversible type of post‐translational protein modification in which LCFAs are covalently linked to cysteine thiol residues in proteins. Different important receptors and signalling proteins in B cells have been identified to be palmitoylated including CD20, CD23 [78], CD81 [79], Fas [80] and HGAL (Fig. 3) [81]. While our knowledge on the functional implications of protein palmitoylation in B cells is currently limited, initial studies have revealed that this type of modification can affect different aspects of B cell function. To illustrate, the palmitoylation of CD81, which is a tetraspanin stabilizing the BCR co‐receptor complex consisting of CD21 and CD19 is palmitoylated. CD81 palmitoylation is necessary for increased BCR‐coreceptor lipid raft association thereby promoting its signalling function [82]. HGAL, which is specifically expressed in normal GC B cells and lymphoma cells derived from the GC, can be myristoylated and palmitoylated. These modifications localize HGAL to raft microdomains and shape BCR signalling by facilitating interaction with SYK (Fig. 3) [83].

Fas palmitoylation has been shown to increase apoptosis. Interestingly, stabilization of HIF1α, a transcription factor central to cellular responses to hypoxia, has been demonstrated to trigger reductive glutamine metabolism and increased Fas palmitoylation [80]. This finding has potential implications for Fas signalling within the GC, as GCs have been reported to harbour hypoxic regions [84].

Altered protein palmitoylation can also be associated with aberrant B cells. For example, palmitoylation of the pro‐apoptotic protein BAX has been found to be reduced in Hodgkin lymphoma B cell lines in comparison to control B cells and correlated with reduced BAX activity [79].

Finally, isoprenoid modifications such as the geranylgeranylation or farnesylation affect the subcellular localization and function of the modified protein. The mevalonate pathway is needed for the generation of isoprenoids, cholesterol and cholesterol derivates [85]. This metabolic pathway requires several key enzymes including HMG‐CoA reductase (HMGCR). In this pathway, mevalonate is synthesized from HMG‐CoA which itself originates from acetyl‐CoA (Fig. 3) [86]. Further processing leads to the generation of farnesyl pyrophosphate (FPP) which is converted to geranyl‐geranyl pyrophosphate (GGPP) or cholesterol. The final products of this pathway, GGPPs, are post‐translational lipid modifications that can alter the activity of important proteins such as the Ras superfamily of small GTPases and as such regulate core cell functions (Fig. 3) [87]. Regulatory B cells, important for restricting inflammatory responses, have been reported to depend largely on GGPPs to drive IL‐10 production through the PI3K‐AKT‐GSK3 axis [88]. Farnesylation occurs by adding a 15‐carbon hydrophobic farnesyl isoprenoid to cysteine residues at the C‐terminus of target proteins such as the GTPases H‐Ras, K‐Ras and N‐Ras. The modification allows the GTPases to anchor to the cell membrane, thereby allowing them to interact with membrane‐associated signalling complexes.

### 3.5 Lipids in redox signalling

Lastly, when considering how lipid metabolism affects B cell fate, it should be noted that lipid catabolism can affect redox balance. Accumulation of certain lipids can induce toxicity and lipid peroxidation is a central step of ferroptosis; an iron‐dependent oxidative form of cell death also reported to be important in B cells [89, 90]. Of note, phospholipids containing PUFAs are vulnerable to peroxidation and thus play an important role in ferroptosis execution (Fig. 3). In contrast, MUFAs are less likely to be oxidized, and incorporation of MUFAs into membrane phospholipids therefore suppress ferroptosis [91].

## 4 Lipids throughout B cell development, differentiation and function

During B cell development and differentiation, B cells display different metabolic requirements perhaps reflecting their level or proliferation, protein secretion, their ability to recycle nutrients via autophagy as well as their localization in metabolically heterogeneous niches. Two highly proliferative B cell subsets exist: B cell precursors (pro‐ and pre‐B cells) and GC B cells. In early stages of B cell development, B cell precursors undergo several rounds of proliferation to increase the population of cells with a functional pre BCR. Similarly, upon stimulation with cognate antigen, activated mature B cells differentiate to GC B cells and proliferate to increase the number of B cell clones able to recognize the invading pathogen. During the humoral immune response, B cells differentiate to PCs, which secrete large quantities of antibodies. Their increased need for protein folding capacity results in a dramatic expansion of the ER network. As discussed above, lipids can play a role as a source of energy, biosynthetic precursor molecules but also affect cell signalling. In the next paragraphs we will focus on different B cell subsets, their general metabolic profiles and the current knowledge on the specific roles of lipids.

### 4.1 Metabolic fate of the early B cell developmental stages

**Fig. 4:**
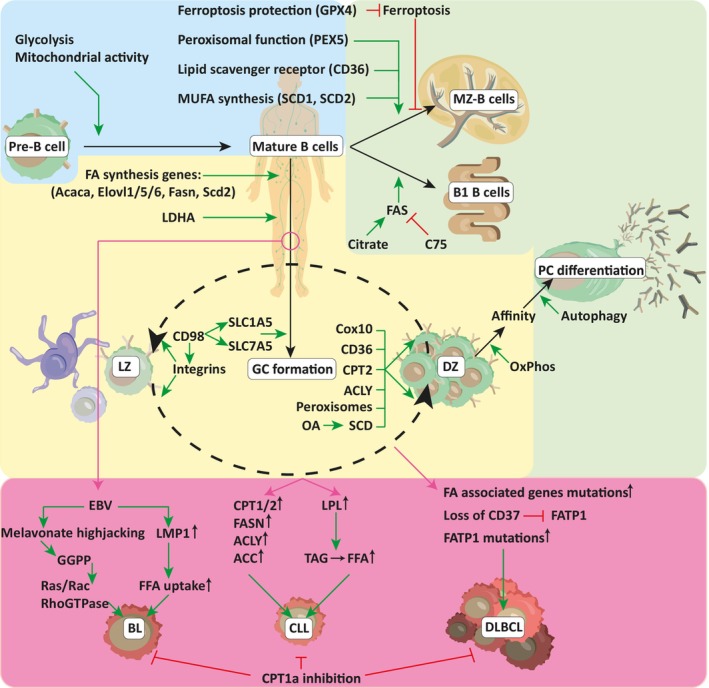
Overview of lipid‐metabolism associated factors in B cell development and malignancies. Throughout the different phases of a B cells life, several proteins and metabolites important for lipid metabolism and homeostasis are involved in maturation, differentiation, and malignant transformation. Initial maturation (Blue segment) of B cells seems to rely mostly on glycolysis and mitochondrial activity, with no major roles for lipids reported. The more innate like MZ‐B and B1 B cells, as well as PCs (Green segment) have been reported to actively take up and process exogenous lipids, which is important for their survival. Activated GC B cells (Yellow segment) seem to require various metabolites including glucose, amino acid and lipids. The exact role for lipids remains ambiguous, yet several reports hint at their importance for GC B cells. Alterations in B cells allowing them to excessively employ lipid metabolism are associated with multiple B cell malignancies (Magenta segment). Abbreviations from top left to bottom right: GPX4: glutathione peroxidase 4, PEX5: peroxisomal biogenesis factor 5, SCD1/2: steraoyl‐CoA desaturase, MZ: marginal zone, FA: fatty acid, Acaca: acetyl‐CoA carboxylase 1, Elovl1/5/6: elongation of very long chain fatty acids protein, Fasn: fatty acid synthase, LDHA: lactate dehydrogenase a, FAS: fatty acid synthesis, SLC1A5/7A5: solute carrier, Cox10: cytochrome C oxidase assembly factor haem A:Farnesyltransferase, CPT1/2: carnitine palmitoyl transferase, ACLY: ATP citrate synthase, OA: oleic acid, LZ: light zone, GC: germinal centre, DZ: dark zone, EBV: Epstein–Barr Virus, LPM1: latent membrane protein 1, GGPP: geranylgeranyl pyrophosphate, FFA: free fatty acid, LPL: lipoprotein lipase, ACC: acetyl‐CoA carboxylase, TAG: triacylglycerol, FATP1: fatty acid transporter protein 1, BL: Burkitt lymphoma, CLL: chronic lymphocytic leukaemia, DLBCL: diffuse‐large B cell lymphoma.

B cells develop from hematopoietic stem cells in the bone marrow and undergo discrete developmental steps during which the immunoglobulin (Ig) heavy‐ and light‐chain loci are sequentially rearranged. Proliferation in early B cell precursors is driven by the IL‐7 receptor and later on by the pre‐BCR, which consists of the rearranged heavy chain and the surrogate light chain [92, 93]. Reportedly, both glycolytic and mitochondrial activity help maintain the highly proliferative character of pre‐B cells receiving optimal signals from their pre‐BCR (Fig. 4) [5, 10, 15, 22, 92, 94, 95]. Little is known about the role lipid metabolism plays in B cell precursors. Loss of peroxisome function in the absence of peroxisomal biogenesis factor 5 (PEX5) does not interfere with early B cell development [96], suggesting that peroxisome dependent FAO is not central to early B cell development. Similarly, mature B cell numbers are unaltered in CD36^−/−^ mice [97]. Lastly, cell intrinsic generation of MUFAs is not essential for B cell development. To generate MUFAs, SFAs are converted by stearoyl‐CoA desaturase (SCD). Although systemic SCD inhibition impinges on B cell development, mice deficient for SCD1 and SCD2 do not display defects in B cell development suggesting that cell intrinsic generation of MUFAs is not required for B cell development [22].

However, since different means of FA uptake exist, it remains unclear whether B cell precursors require external lipids and whether these lipids are used for energy generation or are incorporated into other pathways intracellularly. Similarly, while it is to be expected that proliferating B cell precursors need to generate lipids for membrane synthesis the exact regulation of this process in B cell precursors is currently unknown.

### 4.2 The uncharted metabolic phenotype of mature B cells

Limited knowledge exists regarding the metabolic characteristics of naïve mature B cells. However, it is currently believed that circulating and resting splenic B cells are relatively metabolically quiescent [11] until they undergo activation, at which point mitochondrial and glycolytic activities are upregulated [11, 98]. Interestingly, after activation both an increase in mitochondrial activity and a remodelling of mitochondrial networks have been observed [99]. Mature B cell numbers are not significantly affected by CD36 loss, SCD deletion or PEX5 deficiency suggesting that aspects of lipid metabolism governed by these molecules are not essential to maintain a normal pool of mature B cells (Fig. 4).

In contrast to the majority of mature B cells (also called B2 cells or follicular B cells) innate‐like mature B cell subsets such as marginal zone (MZ) B cells and B1a cells have been shown to be more dependent on lipid metabolism [96]. MZ B cells are a distinct population of mature B cells that resides in the MZ of the spleen and can mount a rapid response to common blood‐borne pathogens without the need for T cell help [100]. Consistent with their ability to quickly respond to stimulation, MZ B cells display an increase in glucose uptake, lactate production and glucose‐derived ATP in comparison to follicular B cells. In addition to an increase in glucose metabolism, MZ B cells also express higher levels of CD36 and take up more lipids than follicular B cells. Consistent with FA metabolism playing an important role in this B cell lineage, MZ numbers are slightly reduced in CD36‐deficient mice as well as in the absence of functional peroxisomes in PEX5‐deficient mice (Fig. 4) [96]. Of note, unlike follicular B cells, MZ B cells require glutathione peroxidase 4 (GPX4) for their survival [89]. GPX4 is a phospholipid hydroxiperoxidase that protects cells from ferroptosis by converting toxic lipid‐associated hydroperoxides to harmless organic alcohols. Thus, an increased rate of lipid uptake might render MZ B cells more prone to ferroptosis and rescue mechanisms need to be in place to prevent cell death.

Similar to MZ B cells, B1a cells in mice represent a distinct B cell lineage with innate‐like properties [101]. B1a cells primarily reside in the lipid‐rich peritoneal cavity and display a BCR‐ repertoire skewed towards common pathogens (Fig. 4). B1a cells are the main producers of the so‐called natural antibodies which are generated even before an infection occurs and provide a first line of protection [102]. B1a cells are large, long‐lived, continuously produce antibodies and maintain their cell numbers by low levels of proliferation [103]. Consistent with their partially activated phenotype, these cells show higher levels of glycolysis as well as OxPhos than resting mature B cells [104]. Unlike B2 cells, B1a cells actively acquire exogenous lipids for storage. Notably, B1a cells display enhanced citrate production under high‐glucose conditions, suggesting that *de novo* lipogenesis is active in these cells alongside uptake of exogenous lipids. Similar to MZ B cells, B1a cell numbers are reduced in the absence of PEX5 and GPX4 [89]. B1a B cells are also highly susceptible to FASN inhibitor C75 [104]. Collectively, these findings indicate that lipid metabolism is intricately connected to the overall fitness and self‐renewal potential of B1a B cells. In summary, B cell subsets with a “pre‐activated” phenotype appear to more readily engage in lipid uptake and processing.

### 4.3 The metabolic landscape of the germinal centre B cell

Upon encountering antigens, B cells can undergo T cell‐dependent or independent activation, inducing proliferation and ultimately differentiation into antibody producing PCs.

B cell activation *in vitro* results in an increase in both lactate secretion as well as oxygen consumption [11]. Moreover, activated B cells increase the expression of genes encoding major mediators of FA biosynthesis such as *Acaca, Elovl1, Elovl5, Elovl6, Fasn*, and *Scd2* (Fig. 4) [105]. FA synthesis is fuelled by glucose acquired from the extracellular space. Inhibition of ACLY, the enzyme which converts mitochondria‐derived citrate to cytosolic Acetyl‐CoA, blocks B cell proliferation and differentiation to PCs suggesting that de‐novo FA synthesis is crucial for B cell expansion [106]. Interestingly, PO and OA, two main MUFAs produced by the enzyme SCD show the highest increase in their relative abundance in activated B cells [22]. In contrast, the abundance of several PUFAs seems to decrease upon activation. Inhibition of SCD has been demonstrated to reduce B cell proliferation and viability, which could be rescued by providing the cells with exogenous OA. Exogenous OA was also able to boost proliferation and class switching of normal B cells [106]. Unlike OA, PA or stearic acid (SA) were not able to promote B cell proliferation and class switch recombination [22]. These findings demonstrate that while OA synthesis is induced in activated B cells, the cells are able to take up exogenous OA as well, which in turn boosts B cell proliferation. The exact fate of the newly generated or acquired FAs is currently incompletely understood, however it has been demonstrated that OA can be oxidized via FAO and that FAO partially supports an OA‐induced boost in proliferation [22].

Lastly, FA synthesis can affect B cell fate in an indirect manner as well. OA has been shown to boost mTOR signalling thereby inhibiting autophagy. Of note, autophagy inhibition has been shown to rescue proliferation of B cells after SCD inhibition suggesting that dysregulated autophagy in response to reduced FA synthesis might negatively impact B cell proliferation [22].

*In vivo*, B cells responding to a peptide antigen seek T cell help and form specialized structures called GCs. GCs serve as hubs for B cell differentiation, SHM of their immunoglobulin genes, affinity‐based selection and ultimately differentiation to memory B cells and PCs [7, 107, 108]. To drive the GC response, a series of intricate cellular and molecular interactions takes place. Gradients of a specific oxysterol (7α,25‐dihydroxycholesterol, synthesized from cholesterol), have been shown to guide B cells towards the B‐T interface and aid in T cell‐dependent B cell differentiation [109]. Following the interaction with helper T cells, various signalling pathways are activated, including CD40 [110] and IL‐4R [111] induced signalling. These signalling events, along with BCR‐mediated phosphoinositide 3‐kinase (PI3K)/Akt activation and subsequent mTORC1 signalling, trigger metabolic reprogramming within the activated B cells. Failure to undergo substantial metabolic reprogramming impedes the normal GC response [112]. Activation through the BCR precedes increased glucose utilization via upregulation of glucose transporter 1 (GLUT1) expression [11]. Activated pre–GC B cells exhibit high levels of glucose uptake and lactate secretion [113, 114] which is again slightly reduced in fully established GC B cells. Glucose metabolism seems to be essential for a successful GC response as treating mice with the inhibitor 2DG reduces GC B cell numbers in a normal as well as an autoimmune setting [115, 116]. Interestingly, deletion of the gene encoding lactate dehydrogenase A (LDHA), an enzyme catalysing the conversion of pyruvate to lactate in resting mature B cells reduces GC formation upon immunization. However, LDHA appears to be less important in established GCs [117].

GC B cells have also been shown to depend on mitochondrial metabolism. Mitochondrial mass, as well as reactive oxygen species (ROS) production, increase when B cells differentiate to GC B cells and B cells die if mitochondrial ATP generation is inhibited with oligomycin or if the component of the electron transport chain (ETC) Cytochrome C Oxidase Assembly Factor Haem A:Farnesyltransferase (Cox10) is deleted (Fig. 4) [118]. Interestingly, the TCA cycle metabolite α‐ketoglutarate has been demonstrated to support GC B cell identity by driving *Bcl6* expression [113]. Moreover, it has been suggested that mitochondrial activity is needed to support a variety of different biological processes in the GC such as redox balance, cellular motility in response to chemokine signalling and actin cytoskeleton dynamics [95] but is perhaps less important to enable proliferation and survival.

These studies demonstrate that shifts in intracellular metabolic pools not only affect energy generation and biosynthesis but can have far reaching effects on cell fate decisions.

In addition to glucose consumption, activation of B cells also leads to a significant increase in amino acid uptake mediated by solute carriers SLC7a5 (LAT1) and SLC1a5 (ASCT2), along with the necessary carrier‐stabilizing chain SLC3a2 (CD98hc) (Fig. 4) [119]. CD98hc was found to be essential for B cell proliferation during GC formation [120] however the amino acid transport function of CD98hc appears to be dispensable for B cell proliferation. Instead, CD98hc is believed to support B cell proliferation via its interaction with integrins [116].

As the GC reaction progresses, coordinated migration of T cells, B cells, and stromal cells leads to the formation of distinct regions within the GC, including the dark zone (DZ) and light zone (LZ) [7, 108]. The DZ is the site of rapid B cell proliferation and SHM to increase BCR affinity [107]. In the light zone germinal centre B cells interact with follicular dendritic cells (FDCs) and T cells and are selected based on their affinity for an antigen. GC B cells unable to recognize an antigen do not receive pro‐survival signals and die by neglect. Since DZ B cells represent the most proliferative population of B cells in the GC it would be intuitive to expect DZ B cells to display the highest levels of metabolic activity. Yet, the expression or activation of metabolic master regulators cMyc and mTORC1, which drive glucose uptake and mitochondrial generation, is restricted to a small subset of LZ‐GC B cells currently interacting with T helper cells [6, 121]. A model has been proposed in which mTOR signalling drives cell growth in the LZ, creating a reservoir of metabolites that is subsequently used up in the DZ. [121] In this manner, the extent of interaction with T cells predetermines the ability of LZ‐GC B cells to cycle in the DZ. Although the exact metabolic profile of LZ and DZ GC B cells remains incompletely understood, analysis of the transcription profile suggests that DZ‐GC B cells favour OxPhos and FAO. In contrast, LZ‐GC B cells display a more glycolytic gene expression profile [118, 122]. Consistent with this notion, LZ‐GC B cells have been found to take up more glucose than DZ‐GC B cells. Of note, OxPhos activity has been found to be increased in high affinity GC B cell clones (See Fig. 4) [118].

The role of lipid metabolism is currently poorly understood in GC B cells. GC B cells have been reported to synthesize FAs as well as to take up FAs from the environment [114]. Interestingly, FA uptake has been demonstrated to increase as the GC reaction progresses. It has been suggested that GC B cells rely on FAO for proliferation. Injection of etomoxir, a CPT1 inhibitor, together with thioridazine, an inhibitor of peroxisomal FAO slightly reduces GC B cell numbers. Similarly, CPT2‐deficient B cells are less competitive in the GC and CD36‐deficiency leads to fewer GC B cells after immunization [97, 114]. However, since CD36 has also been shown to play a role in autophagy, it remains unclear which biological processes require fatty uptake in GC B cells [123]. GC formation is not affected in the absence of PEX5 or GPX4 suggesting that lipid usage and processing might be different in GC B cells and other cells with high lipid uptake such as B1a cells and MZ B cells.

Summarizing, recent years have brought an increased understanding of how metabolism is regulated in the GC, yet many questions remain unanswered. The biological nature of the GC B cell makes it one of the more challenging cell types to study. The GC is very heterogeneous, highly dynamic, and depends on many different cells maintaining structural and organizational integrity [108]. GC B cells are primed to die and quickly lose viability *ex vivo*. Moreover, without the metabolic and signalling cues of the GC microenvironment, it is possible that the nature of the cells quickly changes *ex vivo* rendering metabolic analysis challenging. Nevertheless, combined, the discussed studies suggest that various subsets of GC B cells display different metabolic programs and that metabolites play complex roles within the GC governing not only energy homeostasis but also migration, differentiation and interaction with other cells.

### 4.4 Lipid metabolism in plasma cell differentiation

The final output of the humoral immune response is the generation of PCs. Once a B cell successfully differentiates into a PC, new metabolic challenges arise. PCs need to secrete large quantities of soluble antibodies, necessitating extensive lipid synthesis for the production of membrane lipids in the ER [124]. Simultaneously, antibody glycosylation consumes a significant portion of glucose, limiting its availability as an energy source for ATP production [16]. PCs can reside in different environments such as the spleen, the bone marrow and the lamina propria of the gut and can be short‐ or long‐lived [16]. Both, their localization as well as longevity can shape the respective metabolic profile of different PC subsets [125, 126]. CD28, a receptor best known for its role as a costimulatory mediator on T cells has also been found to be expressed on PCs. CD28 can modify their metabolic program and plays a critical role in their viability and long‐lived phenotype [127]. Engagement of CD28 by dendritic cells in the stromal niche of the bone marrow induces glucose uptake, mitochondrial mass expansion, and mitochondrial respiration in PCs [127]. PCs seem to be able to use FAs for energy generation as treatment with etomoxir, the inhibitor of LCFA import into the mitochondria, reduces oxygen consumption of these cells [126]. Moreover, inhibition of ACLY‐dependent lipogenesis during B cell activation limits PC differentiation [106]. Several studies report on the effect of different dietary FAs on the humoral immune response. PA has been found to boost numbers of IgA producing PCs [128]. Similarly, administration of n‐3 PUFAs has been shown to boost antibody production *in vivo*, and to partially restore antibody production defects upon immunization in the context of obesity [129, 130]. Different and partially contradictory effects of n‐3 PUFAs and n‐3 PUFA‐derived metabolites on B cell activation, PC differentiation and antibody secretion have been reported [21, 131, 132]. The limitation of these studies is that systemic treatment with metabolites affects other cells crucial for the immune response in addition to PCs. Similarly, these studies do not sufficiently differentiate between effects on B cell proliferation, PC differentiation and survival and therefore our understanding of lipid metabolism in PCs remains incomplete. Nevertheless, the observation that dietary fats influence the outcome of the humoral immune response warrants further investigation into the role of these metabolites in the future.

## 5 Lipid metabolism in B cell malignancies

A hallmark feature of cancer cells is their high intake of glucose associated with significant lactate secretion. Glucose metabolism has therefore taken centre stage in many studies focusing on metabolic reprogramming in malignant B cells [133, 134]. However recent work has highlighted the importance of lipid metabolism and revealed new and unexpected roles for lipids in governing survival and proliferation of malignant B cells (Fig. 4).

We have previously uncovered a striking connection between the loss of membrane protein tetraspanin CD37 on mature B cells and an enhanced uptake of FAs from the serum *in vivo* [135]. Mechanistically, CD37 inhibits fatty acid transporter 1 (FATP1) thereby reducing FA uptake. CD37‐deficient lymphoma cells are more aggressive and take up lipids which they store in lipid droplets or use for energy generation as well as biosynthesis. Interestingly, by increasing lipid metabolism, CD37 deficiency creates a metabolic vulnerability as CD37‐deficient cells are more sensitive to CPT1 inhibition than CD37 sufficient cells [135].

Remarkably, when analysing genomic data from a vast number of tumours across various cancer types, genes associated with lipid metabolism were found to be the most frequently altered, indicating their crucial role as oncogenic drivers. Specifically, an overwhelming 97.3% of the examined DLBCL tumours exhibited genetic alterations in lipid metabolism‐associated genes [136]. Moreover, FATP1 has been found to be significantly overexpressed during the malignant progression of lymphoma [137] and increased FA metabolism has been identified as a hallmark of several chemoresistant cancer types [90], including haematological malignancies [138, 139].

Epstein–Barr virus (EBV) transformed B cell malignancies are also associated with increased lipid metabolism. EBV‐induced expression of latent membrane protein 1 (LMP1) results in significant changes to the FA metabolism of Burkitt's lymphoma cell lines *in vitro* [140]. Notably, FA synthesis and lipid droplet formation have been found to increase and the inhibition of lipogenesis greatly diminishes cellular viability.

Interestingly, proteomics of primary human EBV infected B cells revealed that EBV seemingly highjacks the cholesterol and FA pathways of resting B cells in order to transform them into activated lymphoblasts [141]. Mechanistically, the EBV‐infected B cells reroute their mevalonate pathway towards the production of geranylgeranyl pyrophosphate (GGPP) instead of cholesterol. GGPP post‐translationally modifies several cell signalling proteins which are associated with a broad spectrum of cancers such as Ras, Rac and Rho GTPases, thereby greatly affecting their activity [142]. As such, the highjacked mevalonate pathway in EBV‐transformed B cells likely contributes to pro‐survival signalling.

Lastly, unlike in other B cell malignancies the energy supply of chronic lymphocytic leukaemia (CLL) cells depends more on OxPhos than on glycolysis. Moreover, CLL cells are characterized by increased FA utilization. Unlike normal lymphocytes, CLL cells have been found to express lipoprotein lipase (LPL) [143]. LPL catalyses the conversion of triglycerides into free FAs and is needed for the survival of CLL cells but not normal cells [143]. In addition to LPL, CLL cells show an increased expression of several proteins involved in lipid metabolism including CPT1, CPT2, FASN, ACLY, and ACC [144]. Inhibitors of FA metabolism such as CPT1 inhibitors have shown promising results in decreasing CLL cell growth or viability [145]. Similarly, inhibition of CPT1 seems to be a promising tactic against acute myeloid leukaemia [146, 147, 148], Burkitt lymphoma [149], and as mentioned before, DLBCL [135].

In summary, these findings underscore the significance of FA metabolism in the incidence, progression, and severity of B cell malignancies.

## 6 Conclusion and outlook

Accumulating evidence suggests that lipid metabolism plays a variety of roles in B cell fate and function. Yet many questions remain unanswered, owing perhaps to the complexity of the metabolic pathways and the broad spectrum of different lipid species. Lipids can serve as fuel for energy generation as well as building blocks for biosynthesis. Lipids can additionally affect a variety of signalling processes. A future challenge to be taken on in the field is to elucidate how the metabolic and signalling aspects of lipid function intersect. Does membrane fluidity and receptor nanocluster formation change in cells exposed to different external lipids or cells wired to engage in lipid uptake? How is lipid‐dependent post‐translational protein modification regulated in B cells? How do lipid rich microenvironments, dietary changes or obesity impact B cell fate and function? Can we exploit the correlation between lipid metabolism and B cell malignancies? Moreover, how does oxygen availability in the GC and in B cell malignancies affect their ability to utilize lipids? Hypoxia in B cell malignancies might prompt a Warburg effect, prioritizing glycolysis even in oxygenated conditions, potentially reducing reliance on FAO. However, HIF‐1α activation under low oxygen can modulate lipid metabolism genes, impacting lipid synthesis and uptake and potentially prime B cells to thrive on lipids. Lipid raft dynamics, crucial for B cell function, may be altered by changes in lipid composition during hypoxia. Challenges may arise in lipid utilization due to reduced oxygen availability for OxPhos. Answering these questions will provide further insight into how B cell fate is regulated and will allow us to explore new avenues of modulating B cell function in a therapeutic setting.

## Conflict of interest

The authors declare no conflict of interest.

## Author contributions

RP and JJ conceived the project and wrote and edited the manuscript. RP designed and made the figures. [Correction added on 03 April 2024, after first online publication: The Author contribution section has been included in this version.]
